# Death of Dr. Rutherford

**Published:** 1899-04

**Authors:** 


Death of Dr. Rutherford.—Dr. Robert Rutherford, State Health Officer under Ross' administration four years (1886-1889), died at his home in Houston March 31, 1899. He was one of the widest known, and deservedly, most popular members of the Texas medical profession. He was a man of fine social qualities, and had hosts of attached friends all over the South. Dr. Rutherford had long been identified with the quarantine service of the State, and is credited with being the author of the first law enacted by the State for State protection against invasion by epidemic diseases. He was long time health officer of Houston and of Harris county. He was one of the organizers of the State Medical Association, and was a member of that body continuously to the day of his death, having been honored by the Association in many ways. He was made first vice president at the Houston meeting (1885), and at the subsequent meeting at Dallas (1886) he was in the chair a large part of the session. Dr. Rutherford also served four years on the judicial council, and was sent as delegate to the National Medical Association to represent the State Association. He was a loyal friend and a most pleasant, genial companion. Those who knew him best loved him most, and any one could approach him with the assurance of a courteous reception; a uniform politeness and courtesy characterized him. Peace to his ashes. Dr. Rutherford lost his wife only about a year ago. He left no children.

